# S100A11: A Potential Carcinogen and Prognostic Marker That Correlates with the Immunosuppressive Microenvironment in Pan-Cancer

**DOI:** 10.7150/jca.78011

**Published:** 2023-01-01

**Authors:** Xiaozhen Ji, Xin Qin, Xiuming Huang, Wei Wang, Huiyan Li, Chuizhi Zheng, Yanjing Huang

**Affiliations:** 1 Department of Oncology, Hainan General Hospital (Hainan Affiliated Hospital of Hainan Medical University) , Haikou 570311, Hainan, China.; 2 Department of Radiology, Hainan General Hospital (Hainan Affiliated Hospital of Hainan Medical University) , Haikou 570311, Hainan, China.; 3 Department of Thoracic Surgery, Hainan General Hospital (Hainan Affiliated Hospital of Hainan Medical University) , Haikou 570311, Hainan, China.

**Keywords:** Drug resistant, immunosuppressive, S100A11, TCGA, tumor microenvironment

## Abstract

S100 calcium-binding protein A11 (S100A11) has been proved to be an oncogene of most tumors. However, its role in the tumor microenvironment (TME) in pan-cancer stills remains poorly understood. This study used public data from The Cancer Genome Atlas (TCGA) and the Genotype-Tissue Expression (GTEx) database to evaluate the expression of S100A11. The R package “GSVA” was used for Gene set variation analysis (GSVA) of S100A11. The R package “ESTIMATE” was used to further explore the relationship between S100A11 and TME. The Genomics of Drug Sensitivity in Cancer database was used to investigate the effect of S100A11 on the efficiency of anticancer drugs. We found S100A11 expression was upregulated in most tumors and predicted a poor prognosis. Furthermore, S100A11 expression was closely associated with immune regulation-related pathways. Moreover, S100A11 expression in pan-cancer was significantly related to most immunosuppressive cells, such as tumor-associated macrophages (TAM), tumor-associated fibroblasts (TAF), and Treg cells. The expression of S100A11 was significantly related to immunosuppressive genes and immune checkpoints in most tumor types. Additionally, the upregulation of S100A11 expression made patients with cancer resistant to the treatment of most anticancer drugs, such as sorafenib. In brief, our study showed that S100A11 could be used as a potential carcinogen and prognostic marker for most tumor types. The increased expression of S100A11 was closely related to tumor immunosuppressive TME. The upregulation of S100A11 expression made patients with cancer resistant to sorafenib treatment.

## Introduction

S100A11 is a member of the S100 protein family and has high homology with calmodulin and EF-hand calcium-binding proteins, which promotes tumor progression through cell proliferation, metastasis, angiogenesis and immune evasion. Recent studies reported that S100A11 was an oncogene in pancreatic ductal adenocarcinoma 1,2, glioma 3-5, colorectal cancer 6, breast cancer 7, lung cancer 8, thyroid cancer 9, and gastric cancer 10. However, the biological function of S100A11 in the tumor microenvironment (TME) has been rarely reported.

The TME plays a non-negligible role in tumor occurrence and metastasis 11. It refers to the surrounding environment where tumor cells live, and its components include blood vessels, immune cells, stromal fibroblasts, extracellular matrix (ECM), and various signaling molecules 12. The largest number of immune cells in the TME are tumor-associated macrophages (TAMs), which have been reported to be related to worse survival in most tumors 13. TAMs serve as key mediators in the TME by regulating the production of metabolic factors and the distribution of cytokines. Besides TAMs, tumor-associated fibroblasts (TAFs) and Treg cells are immunosuppressive cells that lead to the exhaustion of CD8+ T cells 14-16.

In this study, we analyzed the differential expression, genetic alteration, and potential prognostic value of S100A11 in pan-cancer. We also explored the relationship between S100A11 and TME, including immune pathways, immune cell infiltration, and immune-related genes. We further analyzed the relationship between S100A11 expression and antitumor drug resistance in patients. Our findings revealed the importance of S100A11 in pan-cancer and suggested that S100A11 might have the potential to regulate the immunosuppressive TME.

## Methods

### Data collection

The RNAseq data and clinical information were acquired from the UCSC XENA database (https://xenabrowser.net/datapages/). A total of 10496 samples with RNAseq data were enrolled, including 9784 tumor samples and 712 normal samples. For OS, DSS, DFI, and PFI survival analysis, 9637, 9163, 4909, and 9479 patients with corresponding clinical information were respectively enrolled. The cBioPortal database (https://www.cbioportal.org/) was used for analyzing and exploring the genetic variations of S100A11. The Genomics of Drug Sensitivity in Cancer database (https://www.cancerrxgene.org/) was employed to evaluate the role of S100A11 in antitumor drug resistance. The sample size in this study is presented in Supplementary Table 1. The immune-infiltrating and stroma cells were provided by the TIMER2 database (http://timer.cistrome.org/) and ImmuCellAI database (http://bioinfo.life.hust.edu.cn/ImmuCellAI#!/).

### Prognostic analysis

The univariate Cox regression (UniCox) was conducted using the R package “survival” and “survminer” to evaluate the relationship of S100A11 with the OS, DSS, DFI, and PFI in the TCGA cohort.

### Enrichment analysis

The gene set variation analysis (GSVA) was used to explore the potential role of S100A11 using the R package “GSVA” based on HALLMARK pathways acquired from the MSigDB database (http://www.gsea-msigdb.org/gsea/index.jsp). Gene set enrichment analysis (GSEA) was conducted by R package “clusterProfiler”.

### Tumor microenvironment analysis

The R package “ESTIMATE” was used to determine the scores for stromal, immune, tumor purity, and ESTIMATE score. ESTIMATE is the value of stromal score plus the value of immune score. The TME-related signatures were downloaded, and the scores were computed based on the published findings 17. The correlations between S100A11 and these scores were further analyzed.

### Statistical analyses

All statistical analyses in this study were based on the mean ± standard deviation. The Student t test was used to compare the differences between the two groups. R software (version:4.1.1) was used for statistics and visualization.

## Results

### Expression analysis of S100A11

**Figure 1:**
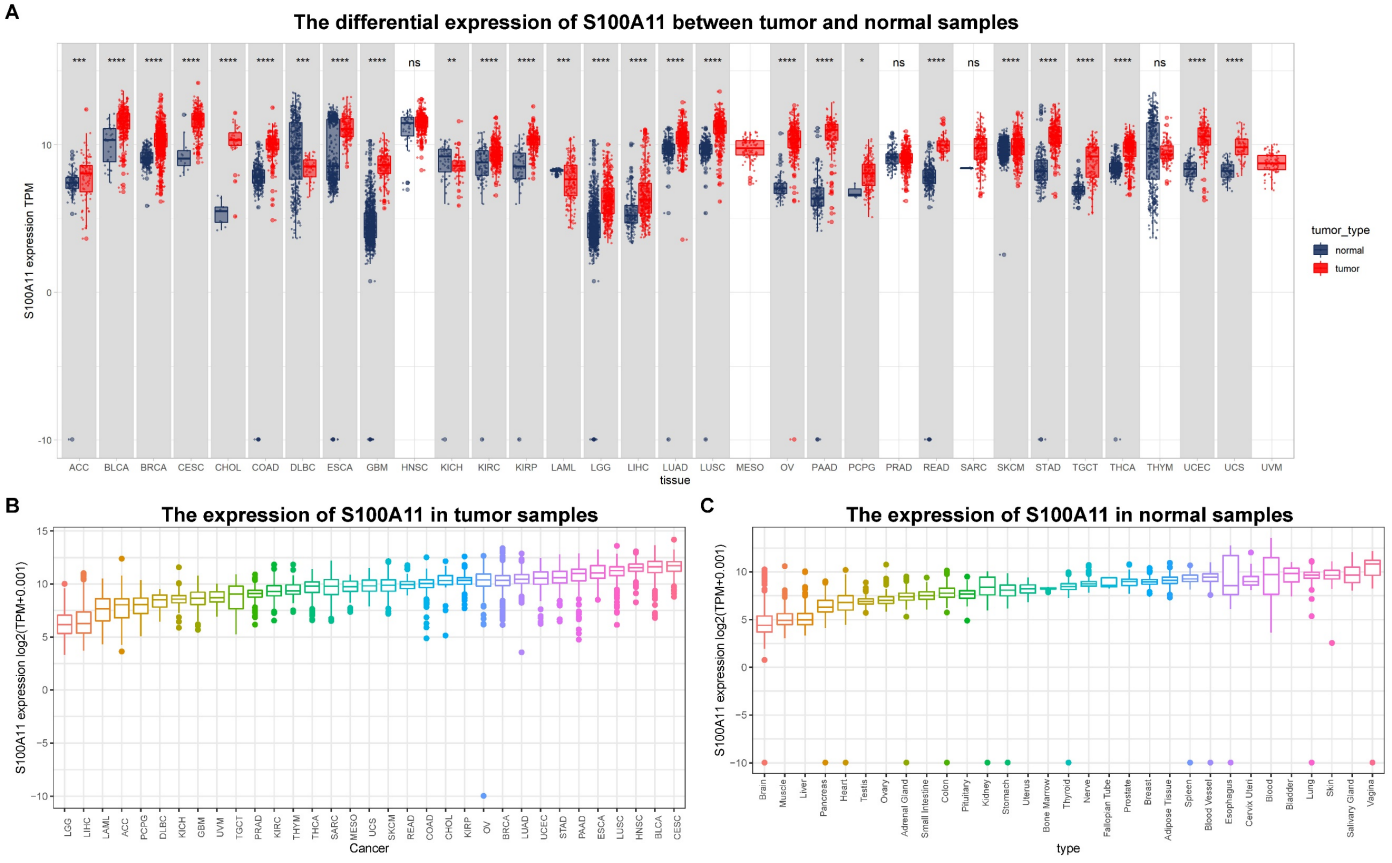
** S100A11 expression in pan-cancer. (A)** Expression of S100A11 based on the TCGA and GTEx databases in pan-cancer. **(B)** Differential expression of S100A11 in tumor tissues in the TCGA cohort. **(C)** Differential expression of S100A11 in nontumor tissues in the GTEx cohort. ^*^P < 0.05, ^***^P < 0.001, ^****^P < 0.0001.

**Figure 2:**
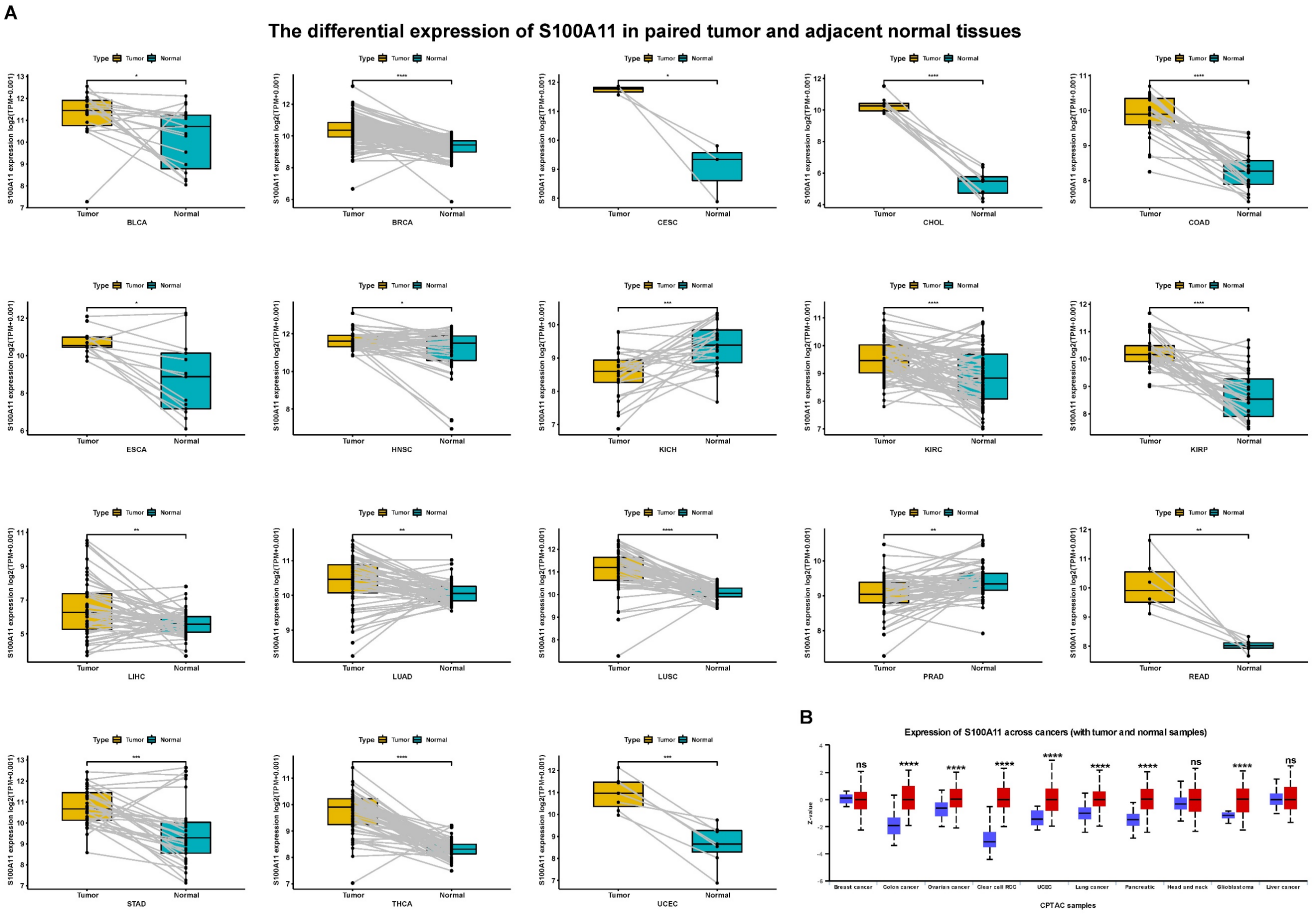
** S100A11 expression in matched tumor tissues and para-cancer tissues. (A)** S100A11 expression in cancer tissues and para-cancer tissues of different types of tumors. **(B)** S100A11 expression in different tumor types compare with normal tissues. ^*^P < 0.05, ^**^P < 0.01, ^***^P < 0.001, ^****^P < 0.0001.

First, we evaluated the expression of S100A11 based on the TCGA and GTEx databases. The results showed that the expression of S100A11 was obviously upregulated in 24 of 33 tumor types, including ACC, BLCA, BRCA, CESC, CHOL, COAD, ESCA, GBM, KIRC, KIRP, LGG, LIHC, LUAD, LUSC, OV, PAAD, PCPG, READ, SKCM, STAD, TGCT, THCA, UCEC, and UCS; however, it was obviously downregulated in DLBC, KICH, and LAML (Figure 1A). S100A11 was expressed only in tumor tissues from the TCGA database, with the highest in CESC and the lowest in LGG (Figure 1B). We also found that S100A11 expression was the highest in the vagina and the lowest in the brain based on noncancer tissue data from the GTEx database (Figure 1C). Single cell data analysis revealed that S100A11 was mainly expressed in malignant cells, macrophages, Tregs, Endothelial cells and fibroblasts (Sup-Figure 1). A comparison of matched tumor tissues and para-cancer tissues in the TCGA database showed that S100A11 expression was upregulated in BLCA, BRCA, CESC, CHOL, COAD, ESCA, HNSC, KIRC, KIRP, LIHC, LUAD, LUSC, READ, STAD, THCA, and UCEC, but downregulated in KICH and PRAD (Figure 2A). Moreover, we also observed that the expression level of S100A11 was obviously upregulated in tumor tissues compared with noncancer tissues in COAD, OV, KIRC, UCEC, lung cancer, PAAD, HNSC, and GBM (Figure 2B).

### Genetic variations of S100A11 in pan-cancer

**Figure 3:**
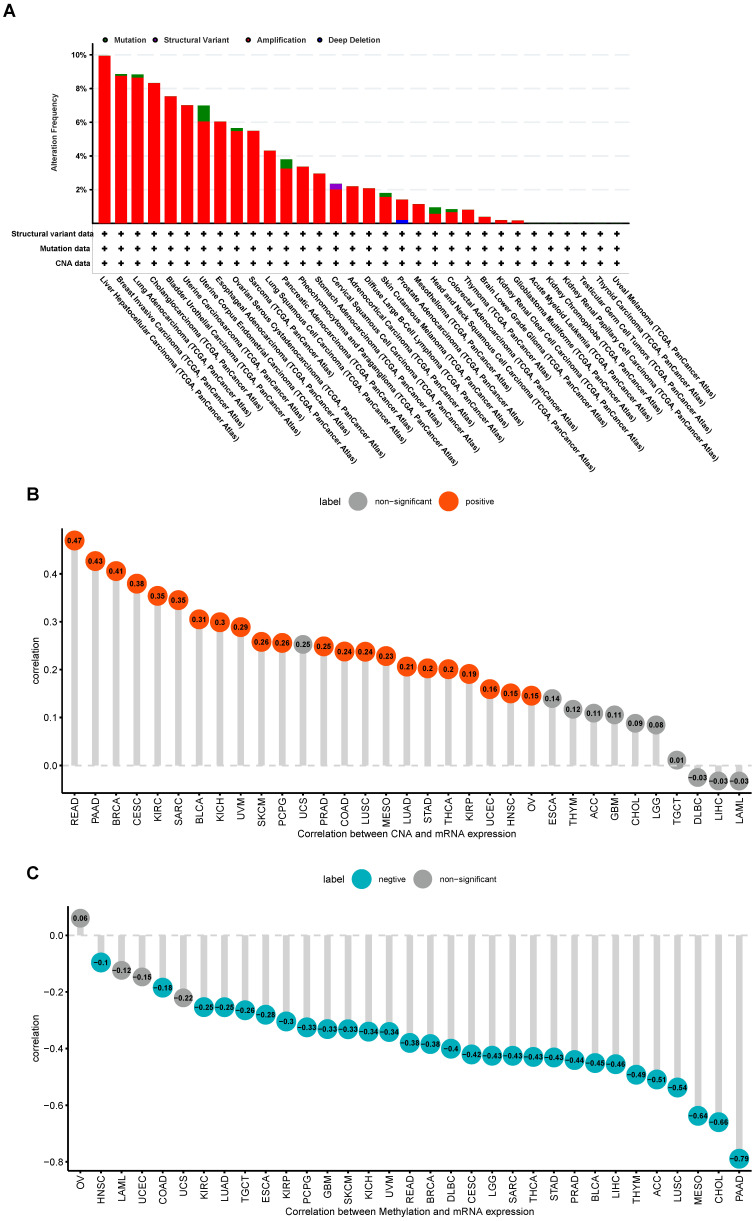
** Genetic variation in S100A11 in pan-cancer. (A)** Gene variation frequency of S100A11 in pan-cancer was analyzed using the cBioPortal database. **(B)** Relationship between S100A11 expression and copy number values. **(C)** Relationship between S100A11 expression and DNA methylation level.

The cBioPortal database was used for exploring the genetic variations of S100A11. The highest alteration frequency of S100A11 appeared in patients with LIHC with “Amplification” as the primary type, as shown in Figure 3A. In addition, S100A11 expression had a positive correlation with copy number alteration (CNA) in 22 of 33 tumor types (Figure 3B) and a negative correlation with the DNA methylation level of S100A11 in 29 of 33 tumor types (Figure 3C).

### Prognostic analysis of S100A11 in pan-cancer

**Figure 4:**
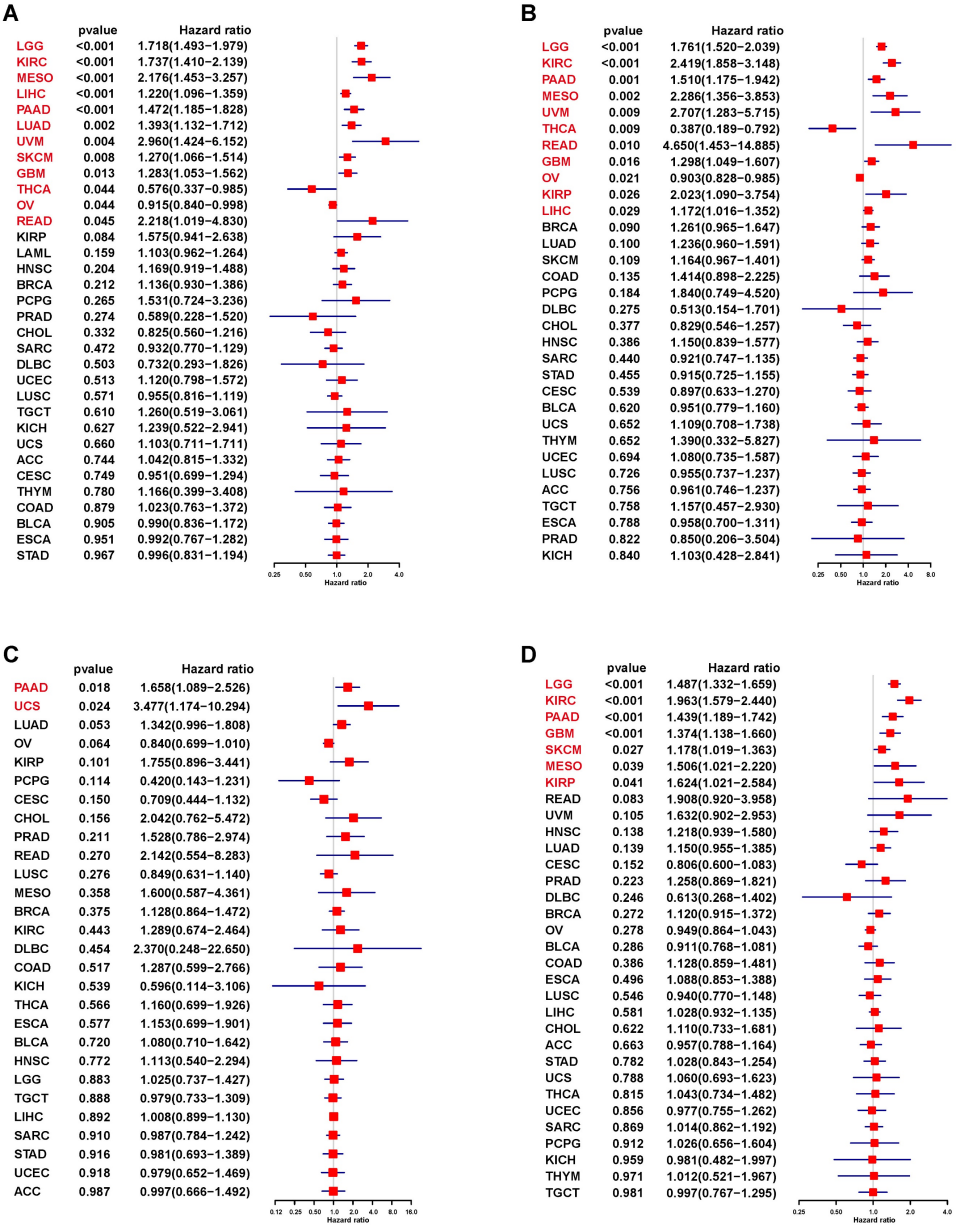
** UniCox analysis of the correlation between S100A11 and prognosis. (A-D)** Forest plot showing the UniCox results of S100A11 in pan-cancer. (A) OS, (B) DSS, (C) DFI, and (D) PFI.

We used UniCox to analyze the correlation between S100A11 and the prognosis of patients with cancer. The UniCox of OS showed that S100A11 was seemly a dangerous factor for LGG, KIRC, MESO, LIHC, PAAD, LUAD, UVM, SKCM, GBM, and READ, and a protective factor for THCA and OV (Figure 4A). The UniCox of DSS showed that S100A11 was seemly a dangerous factor for LGG, KIRC, PAAD, MESO, UVM, READ, GBM, KIRP, and LIHC, and a protective factor for THCA and OV (Figure 4B). The UniCox of DFI showed that S100A11 acted as a dangerous factor for PAAD and UCS (Figure 4C). The UniCox of PFI showed that S100A11 was a dangerous factor for LGG, KIRC, PAAD, GBM, SKCM, MESO, and KIRP (Figure 4D). Kaplan-Meier analysis of S100A11 based on TCGA pan-cancer data also illustrate the risk factor in most tumor types (Sup-Figure 2).

In addition, we validated the prognostic value of S100A11 using Kaplan-Meier Plotter database based on GEO data. Results indicated that high expression of S100A11 indicated worse survival in 8 BRCA datasets, including GSE1456, GSE3494, GSE7390, GSE20685, GSE20711, GSE42568, GSE45255, and GSE65194 (Sup-Figure 3A), as well as LUAD datasets, including CaArray cohort, GSE19188, GSE31210, GSE31908, GSE37745, and GSE50081 (Sup-Figure 3B). CGGA database was used to validate the prognostic value of S100A11 in glioma. Results suggested that S100A11 was a risk factor in glioma based on three independent cohorts (Sup-Figure 3C). The relationship of S100A11 methylation level with OS was also explored. High S100A11 methylation level predicted better OS in LGG, THYM, GBM, and MESO (Sup-Figure 4).

### S100A11 functional analysis

**Figure 5:**
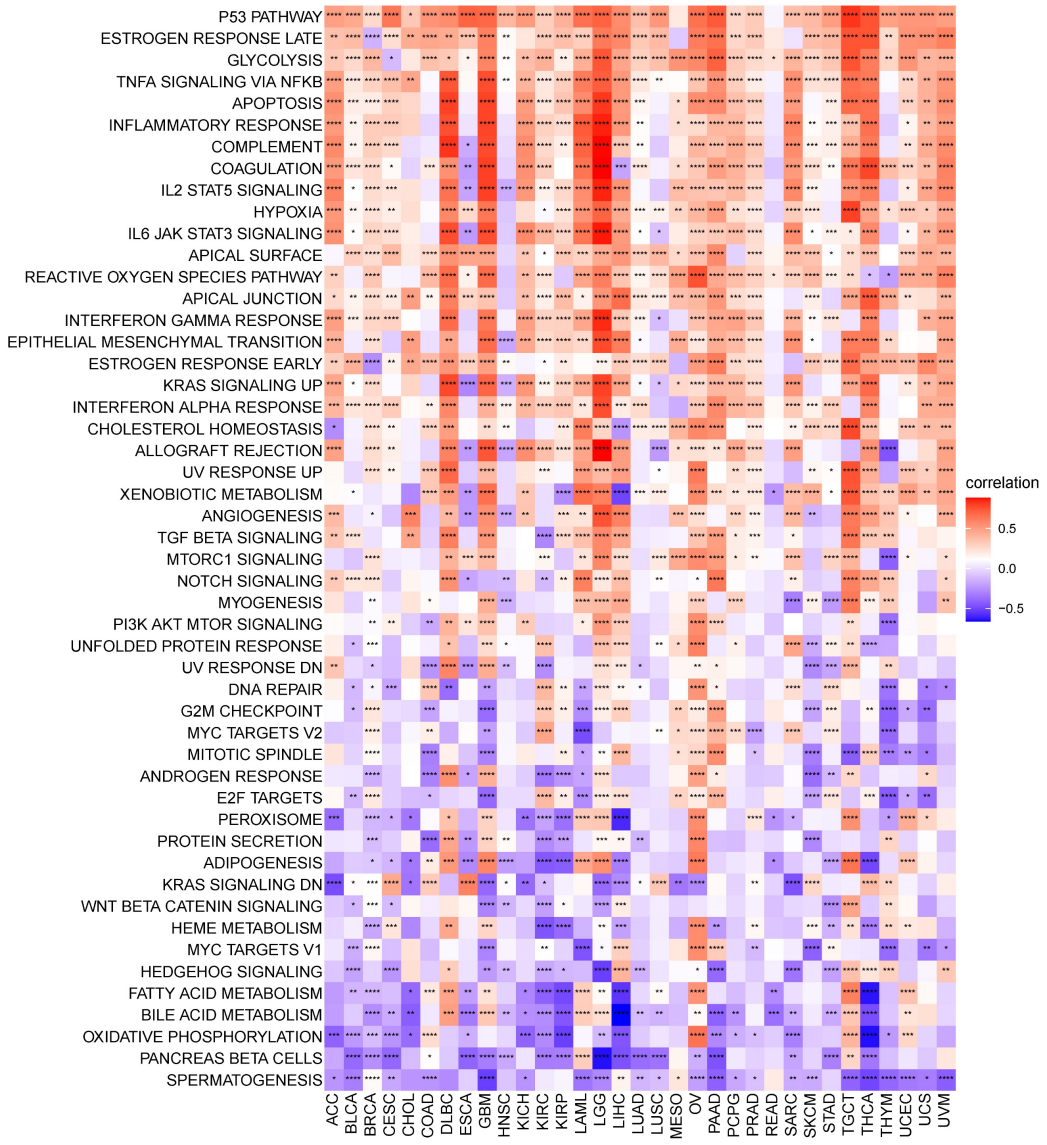
GSVA of S100A11. Correlation between S100A11 and potential pathways in pan-cancer. ^*^P < 0.05, ^**^P < 0.01, ^***^P < 0.001, ^****^P < 0.0001.

We performed GSVA in pan-cancer to analyze the potential pathways with the involvement of S100A11. The relevance of S100A11 expression with GSVA scores is shown in Figure 5. We observed that the expression level of S100A11 in pan-cancer was closely related to many immune response pathways, including P53 pathway, glycolysis, apoptosis, inflammatory response, IL2/STAT5 signaling, hypoxia, and IL6/JAK/STAT3 signaling, suggesting that patients with the elevated expression of S100A11 might be rich in immune cell infiltration. Further GSEA results suggested that S100A11 was closely associated with immune-related pathways (Sup-Figure 5).

### TME analysis of S100A11

**Figure 6:**
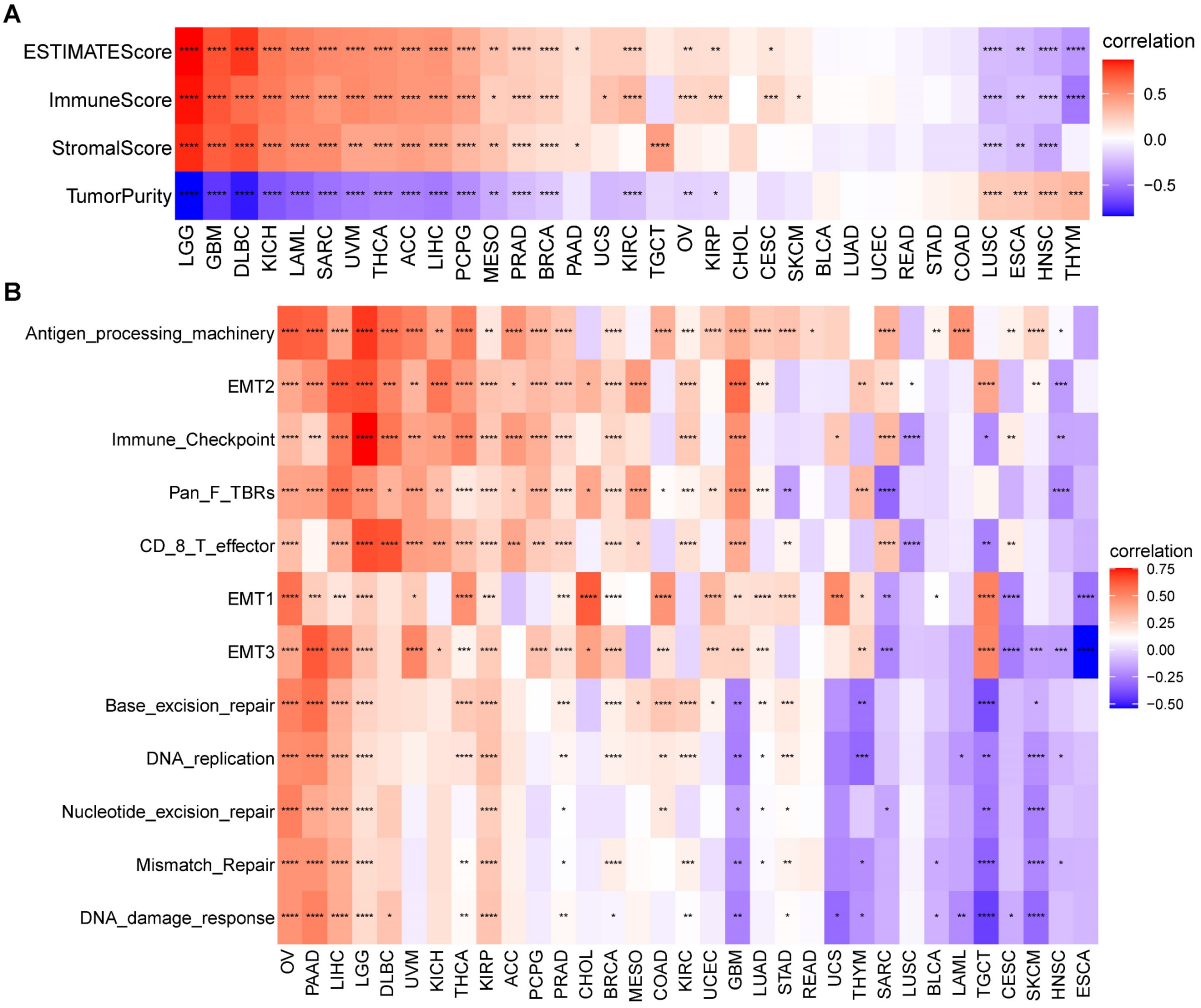
** TME analysis of S100A11. (A)** Heat map represents the correlation between S100A11 expression and immune score, stromal score, ESTIMATE score, and tumor purity score in most tumors. **(B)** Heat map represents the correlation between S100A11 and TME-related pathways. ^*^P < 0.05, ^**^P < 0.01, ^***^P < 0.001, ^****^P < 0.0001.

We next assessed the relationship of S100A11 with TME-related scores. The results suggested that S100A11 positively correlated with immune, stromal, and ESTIMATE scores in most tumors (Figure 6A). In addition, we analyzed TME-related signatures based on the published findings 13, including immune-related signatures, stromal-related signatures, and DNA repair-related signatures. The data analysis showed that S100A11 was closely related to these TME-related pathways in most tumors, particularly for patients with OV, PAAD, LIHC, LGG, and KIRP (Figure 6B).

### Immune infiltration analysis

**Figure 7:**
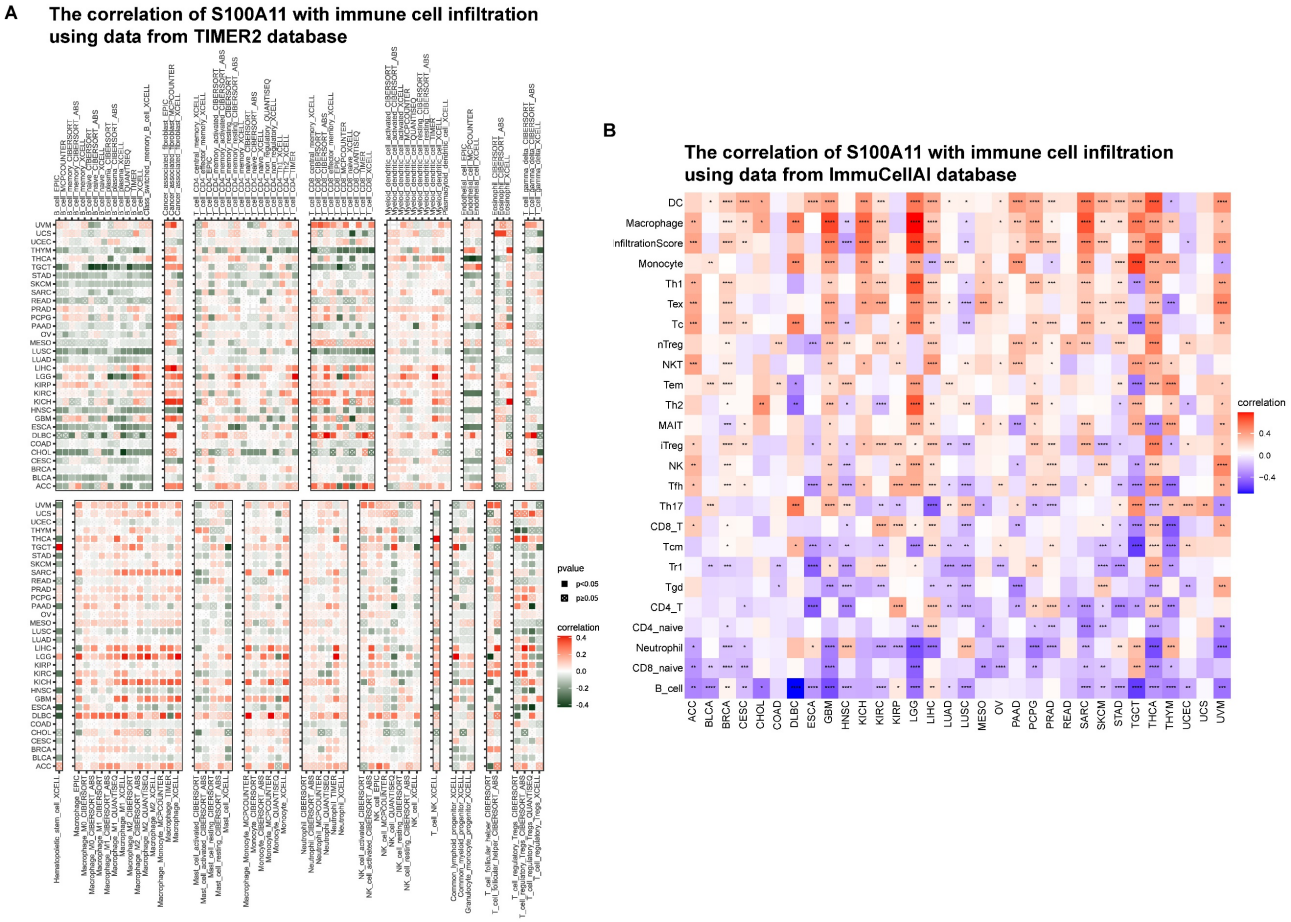
** Immune infiltration analysis. (A)** Relationship between S100A11 and immune cell infiltration in the TME based on the TIMER2 database. **(B)** Relationship between S100A11 and immune cell infiltration in the TME based on the ImmuCellAI database. ^*^P < 0.05, ^**^P < 0.01, ^***^P < 0.001, ^****^P < 0.0001.

Based on the results of the aforementioned analysis, we hypothesized that S100A11 played a key role in the immune microenvironment. Thus, we further analyzed the relevance of S100A11 with immune cells in TME. The immune cell infiltration was analyzed using the TIMER2 database. We discovered that the expression level of S100A11 in pan-cancer was obviously correlated with most immunosuppressive cells, such as TAMs and TAFs (Figure 7A). The immune cell infiltration was analyzed using the ImmuCellAI database, obtaining similar results: immunosuppressive cells, such as TAMs and Tregs, were significantly related to S100A11 expression (Figure 7B).

**Figure 8:**
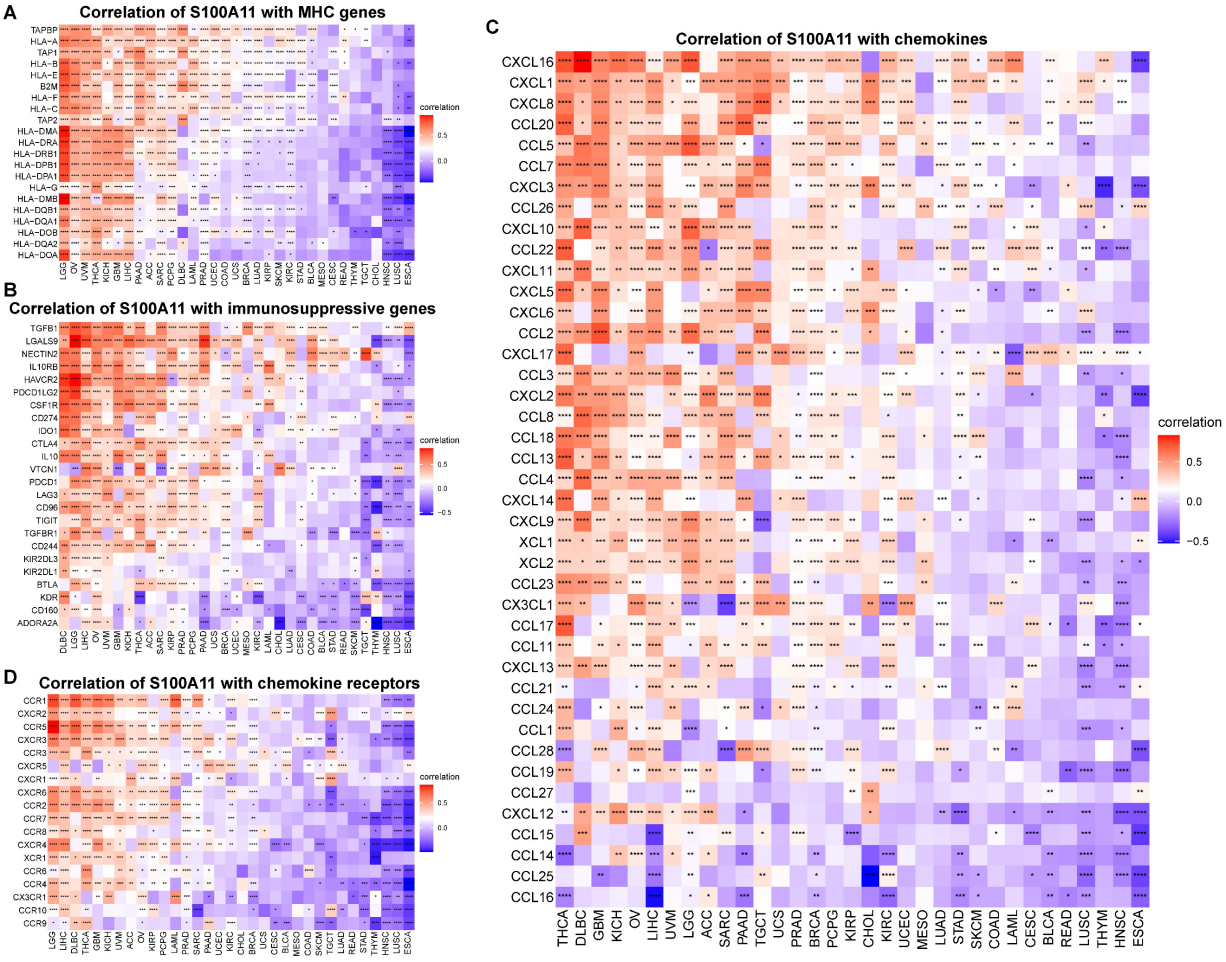
** Correlation between S100A11 and immune-related genes. (A)** Correlation of S100A11 with MHC genes. **(B)** Correlation of S100A11 with immunosuppressive genes. **(C)** Correlation of S100A11 with chemokines.** (D)** Correlation of S100A11 with chemokines receptors. ^*^P < 0.05, ^**^P < 0.01, ^***^P < 0.001, ^****^P < 0.0001.

Additionally, we also observed that S100A11 high expression was significantly related to MHC (major histocompatibility complex) genes (Figure 8A), immunosuppressive genes (Figure 8B), chemokines (Figure 8C), chemokine receptors (Figure 8D), and immune checkpoints (Sup-Figure 6) in pan-cancer, suggesting that patients with increased S100A11 expression might have an immunosuppressive TME. Tgfb1 and Wnt/beta-catenin pathways was associated immunosuppressive microenvironment. We proved that S100A11 was closely associated with genes of Tgfb1 and Wnt/beta-catenin pathways (Sup-Figure 7A, 7B).

### Efficacy analysis of antitumor drugs

**Figure 9:**
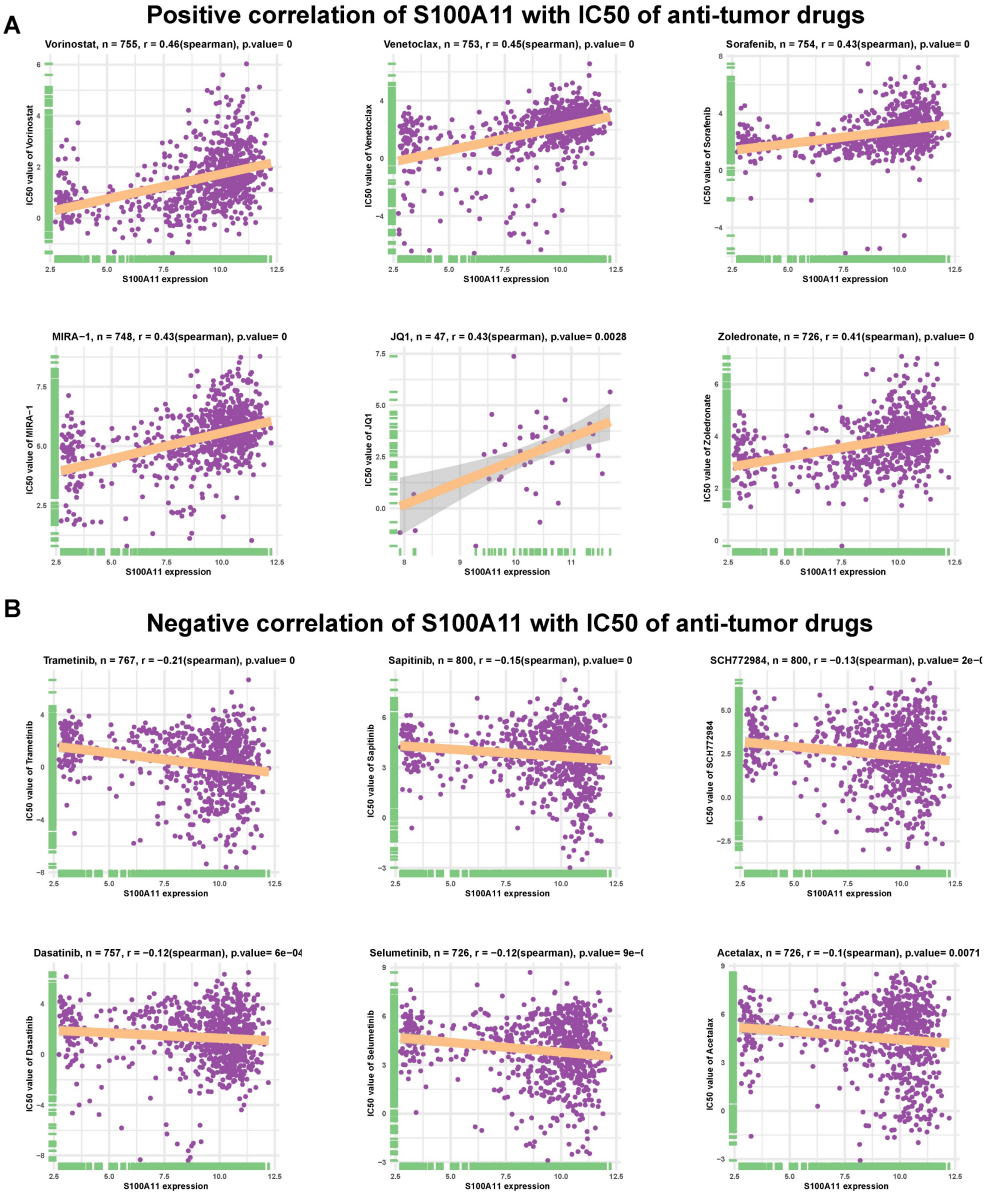
** Efficacy analysis of anti-tumor drugs. (A-B)** Relationship between S100A11 expression and IC50 values of related antitumor drugs. A: Positive results; B: Negative results. *P* value = 0 means *P* value < 0.0001.

We further assessed the association of S100A11 with IC50 of 192 anticancer drugs. The results indicated that S100A11 had a positive correlation with IC50 of 157 of 192 drugs (Supplementary Table 2), such as vorinostat, venetoclax, sorafenib, MIRA-1 (p53 inducer), JQ1 (BET Bromodomain inhibitor), and zoledronate (Figure 9A). These data suggested that the high expression of S100A11 in patients with cancer might lead to drug resistance. Additionally, patients with high expression of S100A11 may be sensitive to the treatment of Trametinib, Sapitinib, SCH772984, Dasatinib, Selumetinib, and Acetalax (Figure 9B).

## Discussion

Studies have shown that S100A11 is expressed in chondrocytes and promotes osteoarthritis progression by activating p38 MAPK 18. Furthermore, the dysregulated expression and cancer-promoting effect of S100A11 have been reported in certain tumor tissues. For example, S100A11 was overexpressed in OV, and the downregulation of S100A11 expression inhibited the invasion and metastasis of ovarian cells 19. S100A11 was overexpressed in small cell lung cancer and suggested the poor survival 20. Nevertheless, its role in the TME has rarely been reported. In this study, we observed that S100A11 was significantly highly expressed in 24 of 33 tumors, including ACC, BLCA, BRCA, CESC, CHOL, COAD, ESCA, GBM, KIRC, KIRP, LGG, LIHC, LUAD, LUSC, OV, PAAD, PCPG, READ, SKCM, STAD, TGCT, THCA, UCEC, and UCS; however, it was significantly lowly expressed in DLBC, KICH, and LAML. The elevated expression of S100A11 predicted worse OS, DSS, DFI, and PFI in patients with cancer, especially in LGG, KIRC, PAAD, and LIHC. Additionally, we discovered that the DNA methylation level of S100A11 was negatively related, while the CNA level of S100A11 was positively related, to S100A11 mRNA expression, implying that the epigenetics of S100A11 might regulate the S100A11 mRNA expression. We further performed GSVA to illustrate the role of S100A11 and found that S100A11 was closely related to many immune-related malignant pathways, such as the P53 pathway, glycolysis, apoptosis, inflammatory response, IL2/STAT5 signaling, hypoxia, and IL6/JAK/STAT3 signaling, indicating that patients with high expression of S100A11 might be rich in immune cell infiltration.

Next, we computed the immune cells and stromal scores of tumors, and discovered that S100A11 expression was closely related to immune cells and stromal scores in most tumors, particularly for patients with OV, PAAD, LIHC, LGG, and KIRP, indicating that S100A11 was highly related to TME immune cell infiltration. Accumulating evidence proved that TAMs and TAFs in the TME were generally remodeled by tumor cells, promoting tumor invasion and metastasis, inhibiting immunity, and stimulating angiogenesis 21,22. We further analyzed immune cell infiltration using TIMER2 and ImmuCellAI databases, and found that the expression of S100A11 was significantly related to most immunosuppressive cells (TAMs, TAFs, and Tregs) in pan-cancer. At present, research of S100A11 is mainly focused on tumor cells, its relationship with immunosuppressive cell, such as TAMs, TAFs, and Tregs, was still unclear. Our work confirmed the expression of S100A11 not only in tumor cells, but also in macrophages, Tregs, Endothelial cells and fibroblasts, indicating a potential function of S100A11 in these immune cells. Besides, our study also observed that S100A11 was significantly related to MHC genes, immunosuppressive genes, chemotactic factors, and chemokine receptors in pan-cancer. The aforementioned data suggested that patients with elevated S100A11 expression might have immunosuppressive TME, eventually contributing to the worse survival status of patients with cancer. These results are novel compared with previous researches.

We also explored the relationship between S100A11 and antitumor drugs, and discovered that S100A11 had a positive correlation with the resistance to most anticancer drugs, such as vorinostat, venetoclax, sorafenib, MIRA-1 (p53 inducer), JQ1 (BET Bromodomain inhibitor) and zoledronate, indicating that patients with tumors with the elevated expression of S100A11 were possibly resistant to these drugs.

In summary, our study proved that elevated S100A11 expression was associated with the immunosuppressive TME in pan-cancer. Targeting S100A11 might activate the immune microenvironment and improve the survival of patients with cancer. We will conduct a series of functional and animal experiments to further verify the role of S100A11 in pan-cancer.

## Supplementary Material

Supplementary figures and tables.

## Abbreviations

ACC: Adrenocortical carcinoma
BLCA: Bladder urothelial carcinoma
BRCA: Breast invasive carcinoma
CESC: Cervical squamous cell carcinoma and endocervical adenocarcinoma
CHOL: Cholangiocarcinoma
COAD: Colon adenocarcinoma
ESCA: Esophageal carcinoma
GBM: Glioblastoma multiforme
KIRC: Kidney renal clear cell carcinoma
KIRP: Kidney renal papillary cell carcinoma
LGG: Lower-grade glioma
LIHC: Liver hepatocellular carcinoma
LUAD: Lung adenocarcinoma
LUSC: Lung squamous cell carcinoma
OV: Ovarian serous cystadenocarcinoma
PAAD: Pancreatic adenocarcinoma
PCPG: Pheochromocytoma and Paraganglioma
READ: Rectum adenocarcinoma
SKCM: Skin cutaneous melanoma
STAD: Stomach adenocarcinoma
TGCT: Testicular germ cell tumor
THCA: Thyroid carcinoma
UCEC: Uterine corpus endometrial carcinoma
UCS: Uterine carcinosarcoma
DLBC: Lymphoid neoplasm diffuse large B-cell lymphoma
KICH: Kidney chromophobe
LAML: Acute myeloid leukemia
HNSC: Head and Neck squamous cell carcinoma
MESO: Mesothelioma
PRAD: Prostate adenocarcinoma
UVM: Uveal melanoma
SARC: Sarcoma
THYM: Thymoma
OS: overall survival
DSS: disease-specific survival
DFI: disease-free interval
PFI: progression-free interval
IC50: half maximal inhibitory concentration
