# Supplementary material for: S100A11: A Potential Carcinogen and Prognostic Marker That Correlates with the Immunosuppressive Microenvironment in Pan-Cancer
Source: J Cancer. 2023 Jan 1;14(1):88–98. doi: 10.7150/jca.78011 (PMC9809332; doi:10.7150/jca.78011)
Supplement: Supplementary file 1 — Supplementary figures and tables. [file jcav14p0088s1.pdf]

Supplementary Figures and Figure legends

Sup-Figure 1

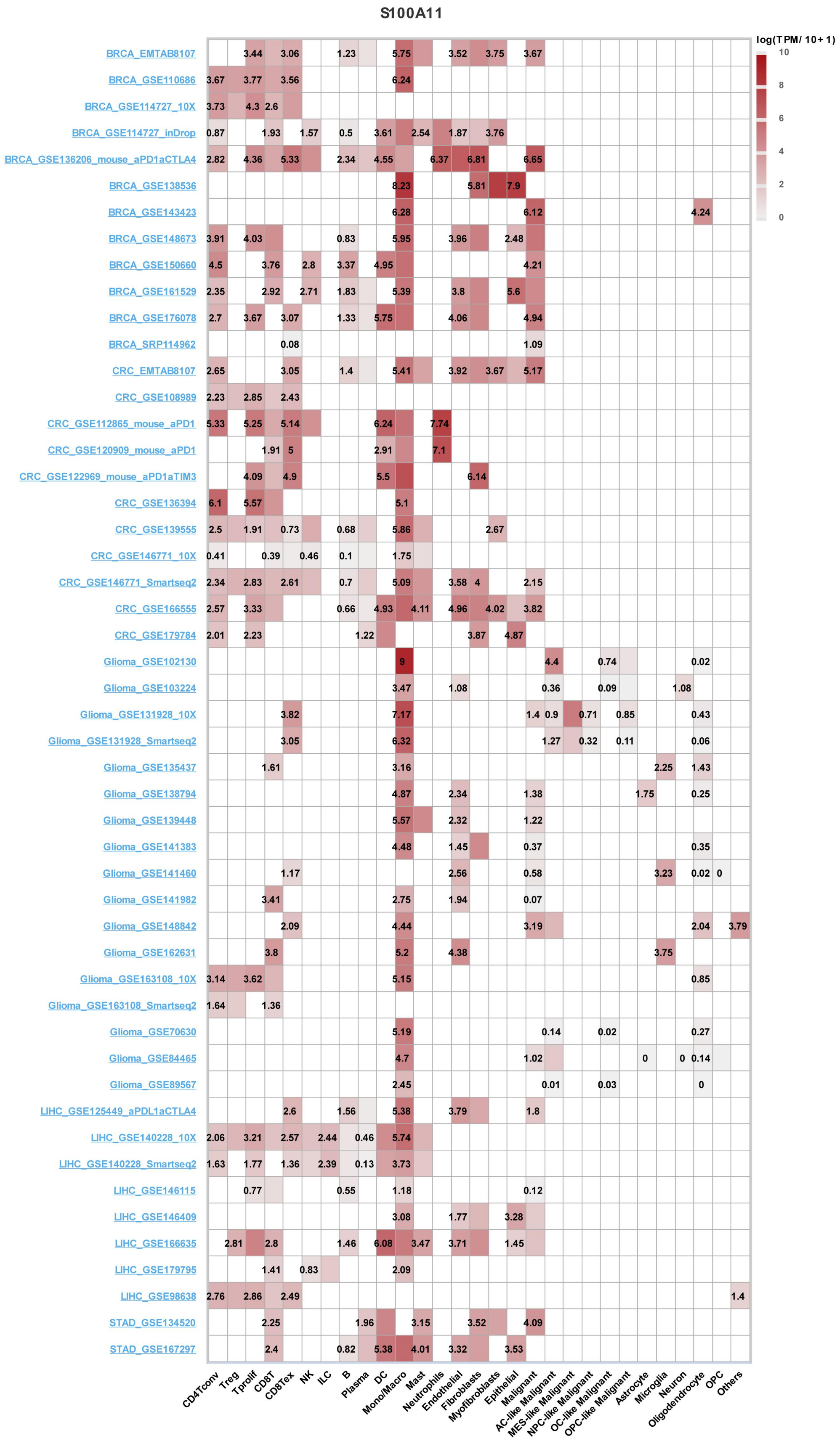

Sup-Figure 1: The single cell expression of S100A11 using TISCH database,

Sup-Figure 2

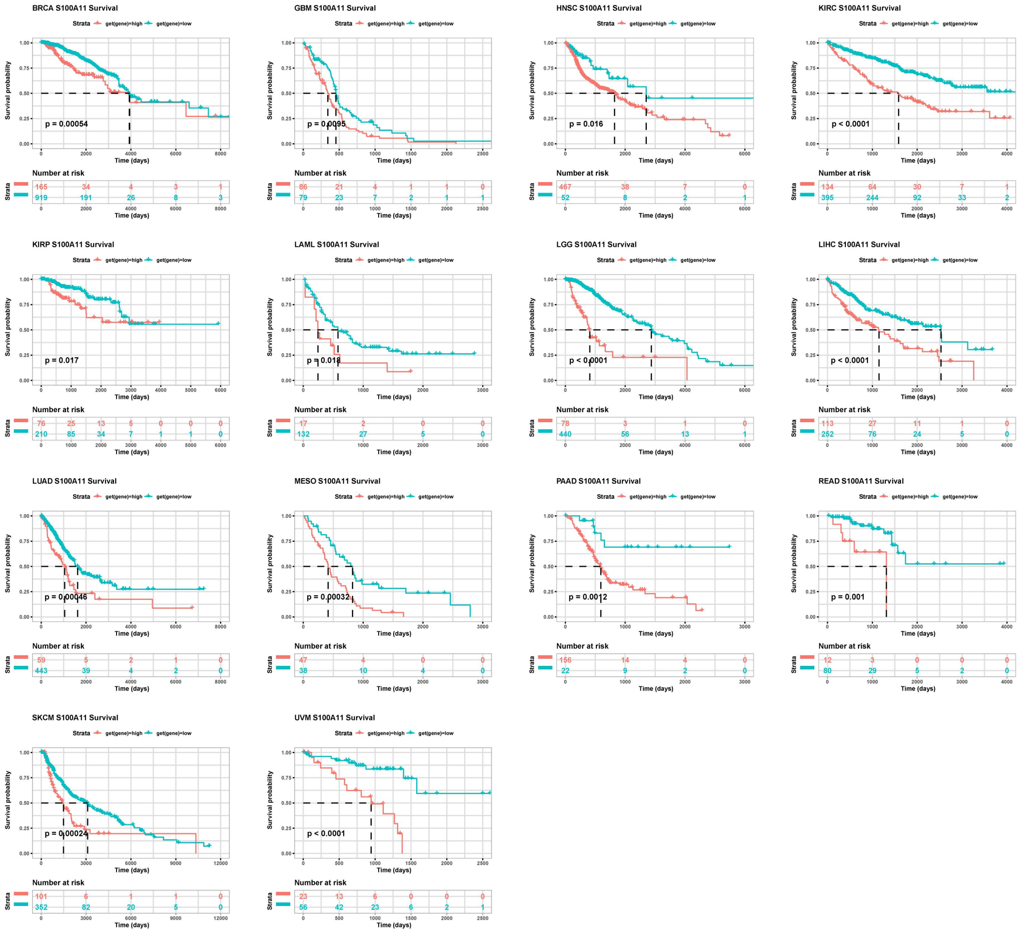

Sup-Figure 2: The Kaplan-Meier curves of S100A11 in TCGA pan-cancer. Only significant results (logrank $p < 0.05$) were displayed.

Sup-Figure 3

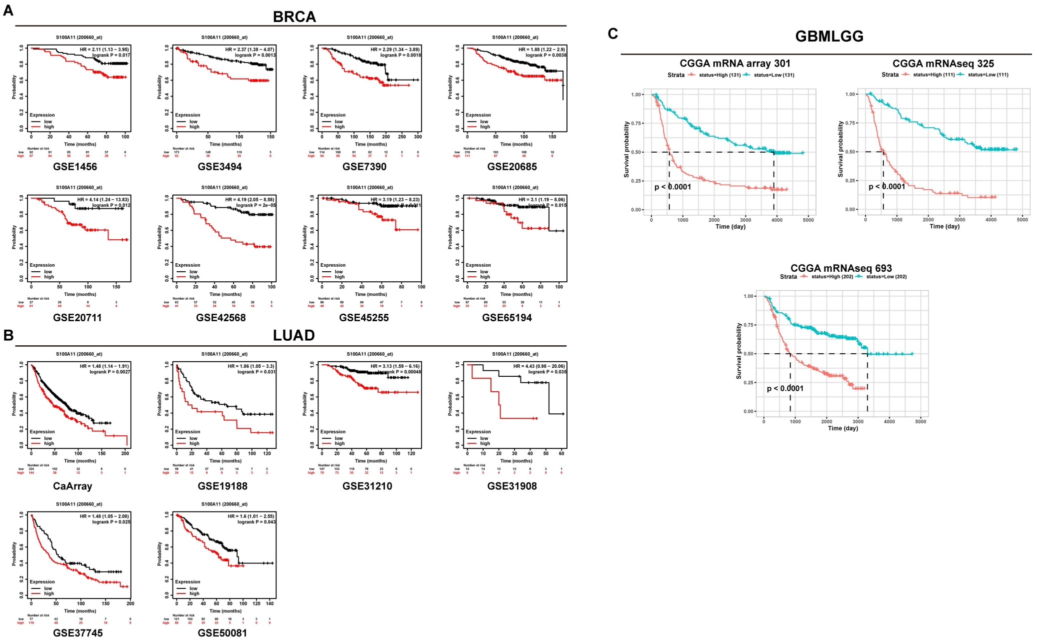

Sup-Figure 3: Survival analysis verification of S100A11.
(A). The survival analysis of S100A11 in BRCA using indicated GEO datasets.
(B). The survival analysis of S100A11 in LUAD using indicated GEO datasets.
(C). The survival analysis of S100A11 in glioma using indicated CGGA datasets.

Sup-Figure 4

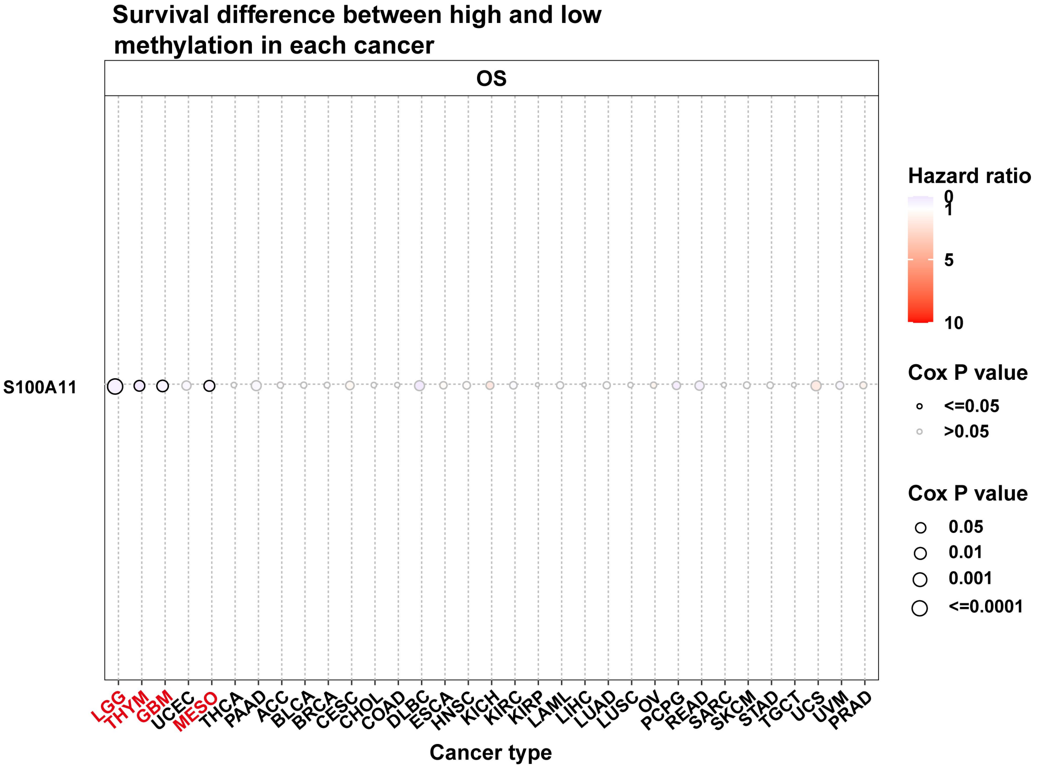

Sup-Figure 4: The survival analysis of S100A11 methylation level.

Sup-Figure 5

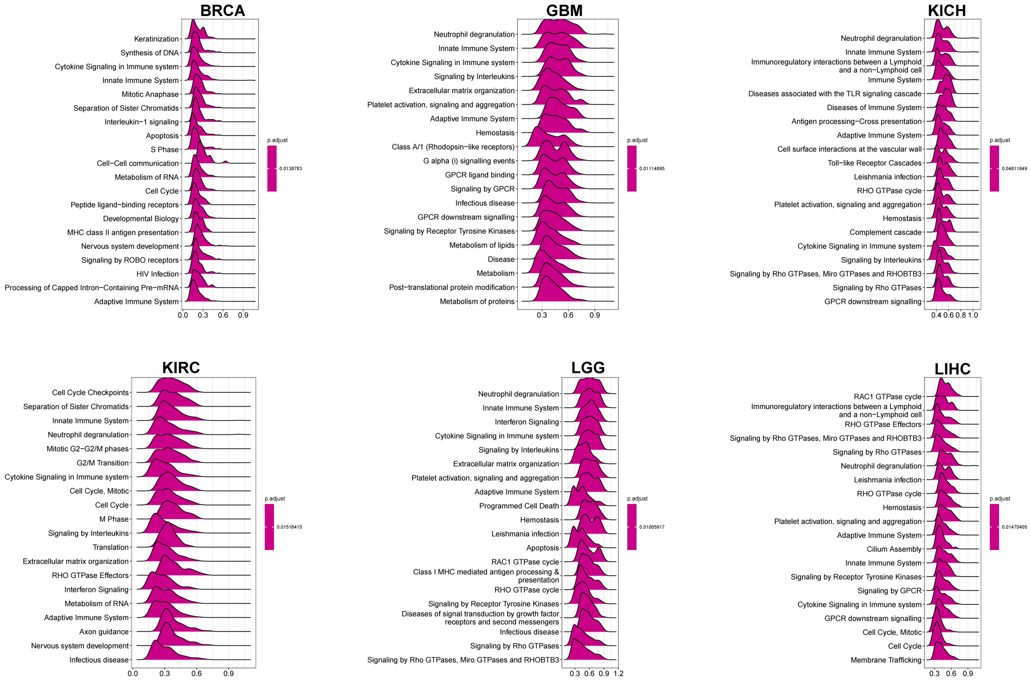

Sup-Figure 5: The top20 GSEA results of S100A11 in indicated tumor types.

Sup-Figure 6

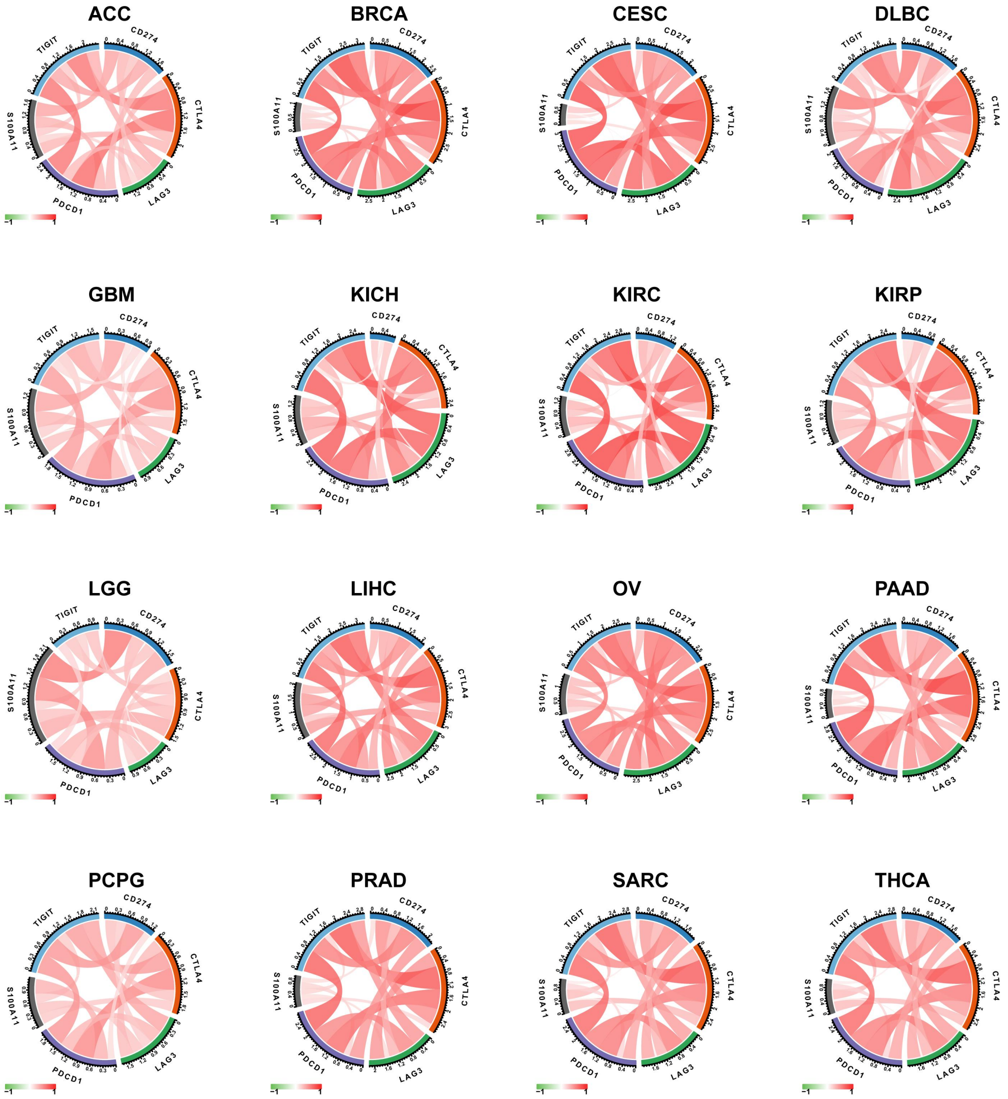

Sup-Figure 6: The correlation of S100A11 with immune checkpoints in indicated tumor types. Red line represent positive correlation. The deeper the color, the stronger the correlation.

Sup-Figure 7

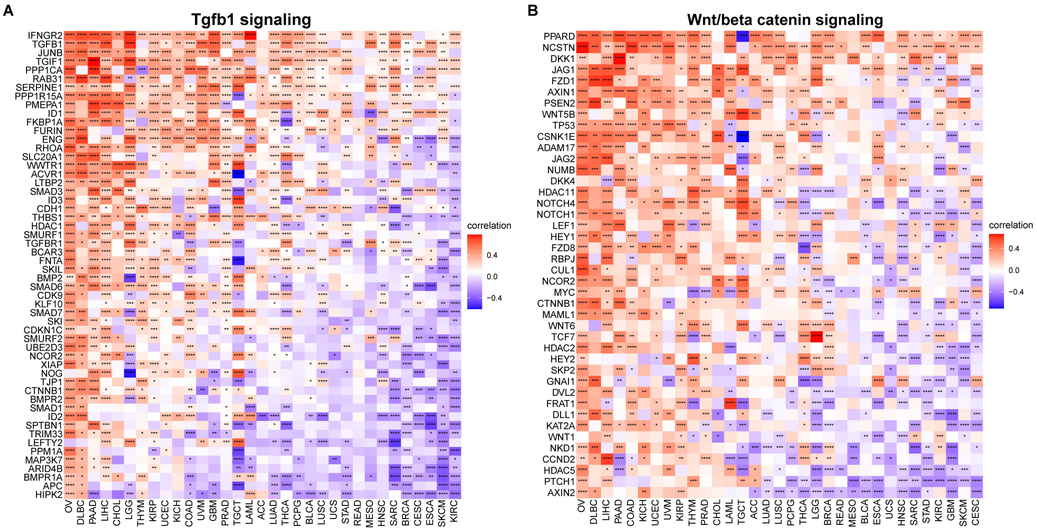

Sup-Figure 7: The correlation of S100A11 with genes in Tgfb1 signaling **(A)** and Wnt/beta-catenin signaling **(B)**.

Supplementary Tables

Supplementary Table 1

Tumor type	Full names	Sample size of tumor tissues from TCGA	Sample size of normal tissues from TCGA	Sample size of normal tissues from GTEx
ACC	Adrenocortical carcinoma	77	0	128
BLCA	Bladder Urothelial Carcinoma	407	19	9
BRCA	Breast invasive carcinoma	1098	113	179
CESC	Cervical squamous cell carcinoma and endocervical adenocarcinoma	306	3	10
CHOL	Cholangiocarcinoma	36	9	0
COAD	Colon adenocarcinoma	288	41	308
DLBC	Lymphoid Neoplasm Diffuse Large B-cell Lymphoma	47	0	444
ESCA	Esophageal carcinoma	182	13	653
GBM	Glioblastoma multiforme	165	0	1152
HNSC	Head and Neck squamous cell carcinoma	520	44	0
KICH	Kidney Chromophobe	66	25	28
KIRC	Kidney renal clear cell carcinoma	531	72	28
KIRP	Kidney renal papillary cell carcinoma	289	32	28
LAML	Acute Myeloid Leukemia	173	0	70
LGG	Brain Lower Grade Glioma	522	0	1152
LIHC	Liver hepatocellular carcinoma	371	50	110
LUAD	Lung adenocarcinoma	515	59	288
LUSC	Lung squamous cell carcinoma	498	50	288
MESO	Mesothelioma	87	0	0
OV	Ovarian serous cystadenocarcinoma	427	0	88
PAAD	Pancreatic adenocarcinoma	179	4	167
PCPG	Pheochromocytoma and Paraganglioma	182	3	0
PRAD	Prostate adenocarcinoma	496	52	100
READ	Rectum adenocarcinoma	92	10	308
SARC	Sarcoma	262	2	0
SKCM	Skin Cutaneous Melanoma	469	1	812
STAD	Stomach adenocarcinoma	414	36	174
TGCT	Testicular Germ Cell Tumors	137	0	165
THCA	Thyroid carcinoma	512	59	279
THYM	Thymoma	119	2	444
UCEC	Uterine Corpus Endometrial Carcinoma	181	13	89
UCS	Uterine Carcinosarcoma	57	0	89
UVM	Uveal Melanoma	79	0	0

Supplementary Table 1: The sample size information

Supplementary Table 2

Correlation	P value	Drug name	Type
-0.123963915	0.000630242	Dasatinib	negtive
-0.209462183	4.72E-09	Trametinib	negtive
-0.145103453	3.80E-05	Sapitinib	negtive
-0.130646524	0.000211094	SCH772984	negtive
-0.12287161	0.000908028	Selumetinib	negtive
-0.099908014	0.007058643	Acetalax	negtive
0.018786032	0.607727369	Gefitinib	non-significant
0.055204082	0.706366593	Axitinib	non-significant
-0.02138515	0.885286001	KU-55933	non-significant
0.213459759	0.149679406	NU7441	non-significant
0.148319457	0.325252222	Doramapimod	non-significant
0.246415356	0.094977554	ZM447439	non-significant
0.253907946	0.081609902	RO-3306	non-significant
0.028154934	0.788778392	AZD8055	non-significant
-0.017442373	0.622069387	PD0325901	non-significant
0.185593476	0.233441035	BI-2536	non-significant
0.090751194	0.539587332	Tozasertib	non-significant
0.101498046	0.492436382	PF-4708671	non-significant
0.013176642	0.718824784	Erlotinib	non-significant
0.165653495	0.260494159	GSK269962A	non-significant
-0.208695652	0.168889457	SB505124	non-significant
0.047645013	0.191260619	Lapatinib	non-significant
-0.000246785	0.994435933	Taselisib	non-significant
0.021887889	0.724362342	OSI-027	non-significant
0.253777367	0.088789288	Ribociclib	non-significant
-0.031123129	0.402395028	ERK_2440	non-significant
-0.06624376	0.074459168	ERK_6604	non-significant
0.071132866	0.055396145	VSP34_8731	non-significant
0.060986641	0.101547013	Ibrutinib	non-significant
0.027137871	0.455930315	AZD3759	non-significant
0.051168317	0.161829418	AZD8186	non-significant
0.062017406	0.089870338	Osimertinib	non-significant
0.026465192	0.47134	VX-11e	non-significant
0.170675301	0.251372011	AZD6482	non-significant
-0.016208598	0.916830301	BMS-754807	non-significant
0.311912233	1.34E-19	Camptothecin	positive
0.381342001	2.87E-27	Vinblastine	positive
0.340473271	3.24E-22	Cisplatin	positive
0.354969378	1.16E-23	Cytarabine	positive
0.228906912	1.86E-18	Docetaxel	positive

0.294751111	1.94E-16	Navitoclax	positive
0.464014955	1.41E-41	Vorinostat	positive
0.352567855	1.73E-23	Nilotinib	positive
0.346505193	8.33E-23	Olaparib	positive
0.240168398	1.97E-11	AZD7762	positive
0.222822357	0.031807046	SB216763	positive
0.073832016	0.036691681	Afatinib	positive
0.086776289	0.016083841	Staurosporine	positive
0.168107831	1.90E-06	PLX-4720	positive
0.362725205	8.44E-25	Wee1 Inhibitor	positive
0.322771683	4.19E-20	Nutlin-3a (-)	positive
0.337847273	7.67E-21	Mirin	positive
0.366689029	1.92E-25	PD173074	positive
0.362522403	5.33E-24	Alisertib	positive
0.240974168	1.35E-11	MK-2206	positive
0.302351353	1.17E-17	Palbociclib	positive
0.173954977	1.67E-06	Dactolisib	positive
0.071852386	0.047250241	Pictilisib	positive
0.218288833	2.79E-09	Obatoclax Mesylate	positive
0.158196481	6.75E-06	5-Fluorouracil	positive
0.288216061	6.86E-16	Paclitaxel	positive
0.292644197	6.10E-16	Crizotinib	positive
0.203182884	2.36E-08	Rapamycin	positive
0.433634227	6.45E-36	Sorafenib	positive
0.360051094	7.80E-26	Irinotecan	positive
0.339065519	2.60E-42	Oxaliplatin	positive
0.072954399	0.045941368	BMS-536924	positive
0.318365936	2.96E-20	GSK1904529A	positive
0.346212134	7.17E-22	PRIMA-1MET	positive
0.404806364	5.31E-30	Niraparib	positive
0.319423933	1.25E-19	MK-1775	positive
0.243380542	3.09E-11	Dinaciclib	positive
0.275714485	1.33E-14	Gemcitabine	positive
0.149374481	3.87E-05	Bortezomib	positive
0.308964801	4.05E-18	Tamoxifen	positive
0.272570399	9.62E-27	Fulvestrant	positive
0.403925547	1.13E-32	EPZ004777	positive
0.374916231	2.07E-26	YK-4-279	positive
0.406997934	1.36E-16	Daporinad	positive
0.337027373	9.64E-21	BMS-345541	positive
0.285883645	4.58E-15	AZ960	positive
0.32457297	9.77E-20	Talazoparib	positive
0.162465548	1.09E-05	XAV939	positive

0.247181515	6.04E-12	Dabrafenib	positive
0.363361174	9.87E-25	Temozolomide	positive
0.273137552	6.90E-14	AZD5438	positive
0.265362067	4.07E-13	IAP_5620	positive
0.094652281	0.010720371	AZD2014	positive
0.401471638	1.87E-29	AZD1208	positive
0.115182018	0.001880316	AZD1332	positive
0.302482788	7.94E-17	Ruxolitinib	positive
0.326963784	2.19E-21	Linsitinib	positive
0.296392709	1.18E-16	Epirubicin	positive
0.353680689	1.84E-23	Cyclophosphamide	positive
0.291377521	4.39E-16	Pevonedistat	positive
0.145651713	1.94E-08	Uprosertib	positive
0.234741919	1.56E-10	LCL161	positive
0.172685515	8.80E-07	Luminespib	positive
0.115752006	0.001030998	Alpelisib	positive
0.377890678	1.73E-28	EPZ5676	positive
0.362555544	6.08E-24	IWP-2	positive
0.381485392	1.45E-26	Leflunomide	positive
0.387724133	1.13E-27	Entinostat	positive
0.331198736	6.21E-22	LGK974	positive
0.258973149	1.37E-12	VE-822	positive
0.19934038	6.08E-08	WZ4003	positive
0.32111962	7.09E-19	CZC24832	positive
0.191056283	2.69E-07	AZD5582	positive
0.230061354	4.32E-10	GSK2606414	positive
0.34771025	4.65E-22	PFI3	positive
0.384766592	4.94E-27	PCI-34051	positive
0.299895988	1.49E-16	Wnt-C59	positive
0.247056645	1.52E-11	I-BET-762	positive
0.296954225	3.17E-16	RVX-208	positive
0.194580915	1.26E-07	OTX015	positive
0.308730258	1.78E-17	GSK343	positive
0.329281521	8.06E-20	ML323	positive
0.107391385	0.003767606	Entospletinib	positive
0.246406493	1.67E-11	PRT062607	positive
0.373319603	2.00E-25	AGI-6780	positive
0.318440716	1.43E-18	Picolinici-acid	positive
0.245481816	2.07E-11	AZD5153	positive
0.269385873	1.61E-13	CDK9_5576	positive
0.291666701	1.10E-15	CDK9_5038	positive
0.345218539	1.23E-21	Eg5_9814	positive
0.227422666	5.70E-10	IRAK4_4710	positive

0.299140062	1.79E-16	JAK1_8709	positive
0.382669203	1.35E-26	AZD5991	positive
0.239828791	6.06E-11	PAK_5339	positive
0.28595013	4.14E-15	TAF1_5496	positive
0.213495872	6.57E-09	ULK1_4989	positive
0.22184901	1.55E-09	IGF1R_3801	positive
0.273103903	6.95E-14	JAK_8517	positive
0.329842884	1.44E-20	AZD4547	positive
0.40837717	1.49E-30	Zoledronate	positive
0.358191579	2.12E-23	Carmustine	positive
0.269987154	1.36E-13	Topotecan	positive
0.344356077	1.22E-21	Teniposide	positive
0.325912484	1.99E-19	Mitoxantrone	positive
0.197654478	2.37E-14	Dactinomycin	positive
0.305426808	3.85E-17	Fludarabine	positive
0.343582296	1.62E-21	Nelarabine	positive
0.378233208	6.63E-26	Vincristine	positive
0.356236896	3.81E-23	Podophyllotoxin bromide	positive
0.178314874	1.38E-06	Dihydrorotenone	positive
0.305969206	3.37E-17	Gallibiscoquinazole	positive
0.348961754	3.24E-22	Elephantin	positive
0.225772956	7.63E-10	Sinularin	positive
0.390541285	8.55E-28	Sabutoclax	positive
0.330782304	5.37E-20	LY2109761	positive
0.259749585	1.17E-12	OF-1	positive
0.205442887	2.33E-08	MN-64	positive
0.328128832	1.10E-19	KRAS (G12C) Inhibitor-12	positive
0.120579281	0.000806487	MG-132	positive
0.185082943	3.38E-07	BDP-00009066	positive
0.260910362	4.03E-13	Buparlisib	positive
0.104621687	5.06E-05	Ulixertinib	positive
0.452808946	2.43E-39	Venetoclax	positive
0.333964403	6.03E-21	ABT737	positive
0.195980254	6.54E-08	Afuresertib	positive
0.325616872	5.85E-20	AGI-5198	positive
0.186667944	2.67E-07	AZD5363	positive
0.275119939	1.78E-14	AZD6738	positive
0.216533874	2.13E-09	Cediranib	positive
0.164157415	6.31E-06	Ipatasertib	positive
0.284696858	1.97E-15	GDC0810	positive
0.093677271	0.011055475	GNE-317	positive
0.346202238	1.63E-22	GSK2578215A	positive
0.378622334	6.09E-27	I-BRD9	positive

0.322309389	1.45E-19	Telomerase Inhibitor IX	positive
0.432948765	1.59E-35	MIRA-1	positive
0.311790294	2.38E-18	NVP-ADW742	positive
0.357457658	5.40E-24	P22077	positive
0.305069173	1.42E-17	Savolitinib	positive
0.266304334	1.26E-13	UMI-77	positive
0.116780524	0.001376608	WIKI4	positive
0.139990204	0.000121155	Sepantronium bromide	positive
0.34461881	2.61E-22	MIM1	positive
0.216257741	2.24E-09	WEHI-539	positive
0.235152753	7.20E-11	BPD-00008900	positive
0.285660772	1.71E-15	Foretinib	positive
0.363798538	9.24E-25	BIBR-1532	positive
0.306642089	9.97E-18	Pyridostatin	positive
0.376254962	1.96E-26	AMG-319	positive
0.338376591	1.93E-21	MK-8776	positive
0.347773793	1.24E-22	Vinorelbine	positive
0.301945743	3.97E-17	LJI308	positive
0.201605238	2.97E-08	AZ6102	positive
0.251152277	3.88E-12	GSK591	positive
0.307946188	9.67E-18	VE821	positive
0.23113227	1.87E-10	AT13148	positive
0.426688252	0.002781689	JQ1	positive

Supplementary Table 2: The correlation of S100A11 with indicated anti-tumor drugs
